# Increased SPARC expression in primary angle closure glaucoma iris

**Published:** 2008-10-20

**Authors:** Jocelyn Chua, Li Fong Seet, YuZhen Jiang, Roseline Su, Hla Myint Htoon, Amanda Charlton, Tin Aung, Tina T. Wong

**Affiliations:** 1 Singapore National Eye Centre, Singapore; 2 Singapore Eye Research Institute, Singapore; 3 Department of Pathology, National University of Singapore, Singapore; 4 Department of Ophthalmology, National University of Singapore, Singapore

## Abstract

**Purpose:**

SPARC (secreted protein, acidic, and rich in cysteine) is involved in extracellular matrix (ECM) organization. The purpose of this study was to evaluate the expression of SPARC in iris tissue from primary angle closure glaucoma (PACG) eyes.

**Methods:**

Iris tissue was obtained from peripheral iridectomies performed during trabeculectomy surgery in nine PACG and 16 primary open-angle glaucoma (POAG) eyes at the Singapore National Eye Centre. Three non-glaucoma control iris specimens were obtained from patients who underwent Descemet’s stripping automated endothelial keratoplasty (DSAEK) procedure. SPARC and collagen I expression were quantified by real-time polymerase chain reaction (PCR). The histological distribution of collagen I and III in the iris stroma was determined using picrosirius red polarization. Density of the iris stromal vasculature was also calculated.

**Results:**

The mean age was 68.9±10.9 years and 65.7±12.2 years in POAG and PACG groups, respectively. The PACG iris expressed SPARC 13.6-fold more and collagen I 5.2 fold more compared to non-glaucoma control iris. The PACG iris also demonstrated 3.3 fold higher SPARC and 2.0 fold higher collagen I expression relative to the POAG iris. The density of collagen I was greater in PACG eyes than in POAG and control eyes (p<0.001). The mean density of iris stromal blood vessels per micron square area was similar in all three groups.

**Conclusions:**

SPARC was significantly increased in the PACG iris. The data suggest that SPARC could play a role in the development of PACG by influencing the biomechanical properties of the iris through a change in ECM organization.

## Introduction

Primary angle closure glaucoma (PACG) is a major form of glaucoma in Asia [1]. The condition is visually destructive and has been estimated to cause more blindness than primary open-angle glaucoma (POAG) worldwide [2]. Several anatomic risk factors for the development of PACG have been identified including a shallow anterior chamber depth and short axial length. Simulated model systems have provided evidence that the presence of both increased lens curvature and a short zonule-iris distance contribute to a pupil block in angle closure [3]. Furthermore, both anteriorly located ciliary bodies [4] predispose to non-pupillary block mechanisms in angle closure. One of the anatomic changes that define PACG is the formation of peripheral anterior synechiae (PAS). Synechial angle closure occurs in some but not all eyes with PACG, and it is not clear why only some but not all eyes with narrow ‘occludable’ angles go on to develop PACG. One possibility is that the iris is biologically different in PACG eyes, and this structural difference plays a role in the pathogenesis of PACG.

Previous studies have demonstrated the presence and the relative compositions of collagens I and III in normal iris stroma [5,6]. In PACG eyes, an overall increase in collagen deposition as well as an increase in collagen I relative to collagen III deposition have been reported [7]. An increased inflammatory cellular aggregation in the iris has also been described following acute angle closure attack, which is likely to further contribute to the observed increased collagen I in PACG irides [7].

SPARC (secreted protein, acidic, and rich in cysteine) is a highly evolutionarily conserved prototypic 32 kDa matricellular glycoprotein that is centrally involved in extracellular matrix (ECM) remodeling [8]. SPARC expression is increased during embryogenesis, tissue injury, and tissue repair [9,10]. SPARC features prominently in the development of tissue fibrosis with subsequent loss of organ function such as in the liver, lungs, kidneys, and skin [11-14]. SPARC therefore plays an important role in the development of diseased states. This is corroborated by the creation of SPARC null mice, which displayed several phenotypic abnormalities associated with aberrant ECM structure and assembly [10,15,16]. In particular, adult SPARC null mice exhibited decreased amounts of collagen in the skin and bone [17,18], and fibrotic deposition of collagen was diminished in the absence of SPARC [10]. Hence, increased expression of SPARC is associated with collagen I production while the lack of SPARC results in decreased collagen accumulation. All these data strongly implicate SPARC as a key regulator of collagen incorporation in tissues. In fact, SPARC has long been considered a chaperone for promoting collagen folding, secretion, maturation, and assembly into macromolecules in the ECM [19,20].

In the eye, SPARC expression has been detected in retina ganglion cells and astrocytes where it serves to maintain retinal function [18-21] in the trabecular meshwork [22]. SPARC also has a role in the development of cataract and posterior capsule opacification [23,24]. However, we are still unaware of previous research on SPARC expression in the iris, particularly in relation to PACG. In view of the established role of SPARC in the promotion of fibrotic collagen I deposition as well as the observed increased expression of collagen I previously reported in PACG iris, we performed experiments to evaluate the expression of SPARC in the PACG iris. In addition, we compared the density of iris stromal vascularity to determine whether blood vessel density contributes to observed features in PACG iris ultrastructure.

## Methods

### Study population

This study was conducted in accordance with the Declaration of Helsinki and after approval from the local institutional review and ethics board at the Singapore Eye Research Institute (SERI). Eligible subjects with poorly controlled PACG or primary open-angle glaucoma (POAG) undergoing trabeculectomy surgery were recruited at the Singapore National Eye Centre (SNEC) over a six-month period. An informed written consent pertaining to the donation and storage of human tissue was obtained from all subjects.

POAG and PACG were diagnosed in the presence of glaucomatous optic nerve head damage (defined by the presence of neuroretinal rim loss of the optic nerve head and vertical cup-disc ratio of 0.7 or greater) with corresponding visual field loss. Eyes with PACG had gonioscopic findings of angle closure (present when the trabecular meshwork was not visualized in at least two quadrants on indentation gonioscopy with the presence of PAS). Trabeculectomy surgery was indicated in the presence of poorly controlled glaucoma where there was suboptimal intraocular pressure (IOP) control despite maximal compliant medical therapy and/or progressive visual field loss and optic disc cupping. A combined trabeculectomy and cataract surgery was performed in some cases where clinically indicated. Relevant preoperative data collected includeed age, gender, race, glaucoma type, history of acute angle closure attacks (if any), prior laser iridotomy or iridoplasty, preoperative IOP, and glaucoma medications.

Non-glaucomatous control subjects comprised of patients undergoing Descemet’s stripping automated endothelial keratoplasty (DSAEK) procedure for the management of corneal endothelial decompensation, which is secondary to Fuchs endothelial dystrophy. Patients with pre-existing glaucoma or those with corneal decompensation as a result of intraocular inflammation were excluded.

### Iris specimen collection

The iris tissue specimens were obtained from peripheral iridectomies performed as part of a standard trabeculectomy and DSAEK procedure. Intraoperatively, the peripheral iridectomies were performed in the superior peripheral iris between 10 and 2 o’clock. Each iris tissue specimen obtained was fixed in a test tube containing 3% paraformaldehyde and subsequently embedded in paraffin wax. All specimens were cut for histological staining and reverse transcriptase polymerase chain reaction (RT–PCR) analysis.

### Polarization microscopy using Sirius red stain

Iris samples were stained with picrosirius red F3BA (Sigma Sirius Red; Sigma, Steinheim, Germany) stain. Qualification of the amount of collagen I present using polarized light was performed using a grading system of 1–4, which estimated the percentage of the section that was stained red: grade 1 (25% or less), grade 2 (25%–50%), grade 3 (50%–75%), and grade 4 (75%–100%). Due to the subjectivity of the grading, each section was graded by two masked independent medical personnel (T.T.W. and A.C.) where inter-observer variability was assessed. To ascertain the validity of our findings, real-time RT–PCR was performed on all the specimens to provide a quantitative analysis, which would support the histopathological assessment.

### Density analysis of iris stromal vasculature

The paraffin sections were stained with hematoxylin and eosin (H&E) and examined using light microscopy. For each iris section, the number of blood vessels was counted manually at 10X magnification, and the cross-sectional area was calculated using an Image J Image Processing and Analysis in Java version 1.40 (NIH Image, Research Services Branch, National Institutes of Health, Bethesda, MD). The density of iris stromal vascularity was derived from the quotient of total number of blood vessels over total cross-sectional area of iris.

### Quantitative real-time polymerase chain reaction

Quantification of SPARC and collagen I expression was performed using real-time RT–PCR. Total mRNA from each group was extracted and purified using the High Pure RNA Paraffin Kit (Roche, Mannheim, Germany) according to the manufacturer’s protocol. cDNA was then synthesized using SuperScript III (Invitrogen, Carlsbad, CA). This was followed by PCR amplification with Power SYBR Green PCR Master Mix (Applied Biosystems, Warrington, UK). The cycling conditions were as follows; denaturation at 95 °C for 10 min followed by 40 cycles at 95 °C for 15 s, 50 °C for 2 min, and 60 °C for 1 min in a LightCycler 480 (Roche). The fluorescent threshold was calculated using the system software, and results analyzed using the comparative cycle threshold method. Collagen I was amplified with the following primers: (forward) 5′-CAG CCG CTT CAC CTA CAG C-3′ and (reverse) 5′-TTT TGT ATT CAA TCA CTG TCT TGC C-3′. SPARC was amplified with the following primers: (forward) 5′-GTG CAG AGG AAA CCG AAG AG-3′ and (reverse) 5′-TGT TTG CAG TGG TGG TTC TG-3′. Normalization was done for each sample against similarly amplified β-actin: (forward) 5′-CCA ACC GCG AGA AGA TGA-3′ and (reverse) 5′-CCA GAG GCG TAC AGG GAT AG-3′.

### Statistical analysis

Statistical analysis of continuous variables was performed using non-parametric Mann–Whitney U test while categorical variables were analyzed using the Student’s *t*-test. A Bland Altman statistical test was used to evaluate for any significant inter-observer variability. A p value less than 0.05 was considered statistically significant.

## Results

### Patient demographic and preoperative data

Twenty-eight subjects were recruited in this study, and the recruits were composed of nine PACG patients, 16 POAG patients, and three non-glaucoma controls. The demographics and relevant clinical details of the glaucoma subjects are shown in Table 1. The mean ages of the POAG and PACG groups were 68.9±10.9 and 65.7±12.2 years, respectively, and the majority of subjects were Chinese (PACG 89% and POAG 88%). The mean number of medications used in both PACG and POAG groups were comparable with the use of topical prostaglandin analogs in 56% of patients in both glaucoma groups. The mean preoperative IOP in the POAG group was 21.7±6.6mmHg and 17.6 ± 3.7 mmHg in the PACG group. The mean vertical cup-disc ratios in the POAG and PACG groups were 0.9±0.07 and 0.8±0.2, respectively. Only two of the nine PACG eyes had a previous history of an acute angle closure episode. All but one eye in the PACG group had undergone laser peripheral iridotomy before trabeculectomy. None of the PACG eyes underwent laser iridoplasty.

**Table 1:** Demographic and preoperative data of POAG and PACG patients.

**Glaucoma Type**	**PACG (n=9)**	**POAG (n=16)**	**p value (2 sided)**
**Mean age in years (SD)**	65.7 (12.2)	68.8 (10.9)	0.46
**Gender**			0.43
Male	5	6	
Female	4	10	
**Race**			1
Chinese	8	14	
Malay	1	2	
Indian	0	0	
Others	0	0	
**Number of eyes with history of acute angle closure**	2	NA	NA
**Mean number of medications (SD)**	1.89 (1.17)	1.95 (0.85)	0.93
**Number of eyes using topical prostaglandin analog (%)**	5 (56%)	9 (56%)	1
**Mean preoperative intraocular pressure (SD)**	17.6 (3.7)	21.7 (6.6)	0.09
**Number of eyes with prior laser iridotomy**	8	NA	NA
**Mean cup-to-disc ratio (SD)**	0.8 (0.2)	0.9 (0.07)	0.09

### mRNA expression of SPARC and collagen I in the iris

**Figure 1:**
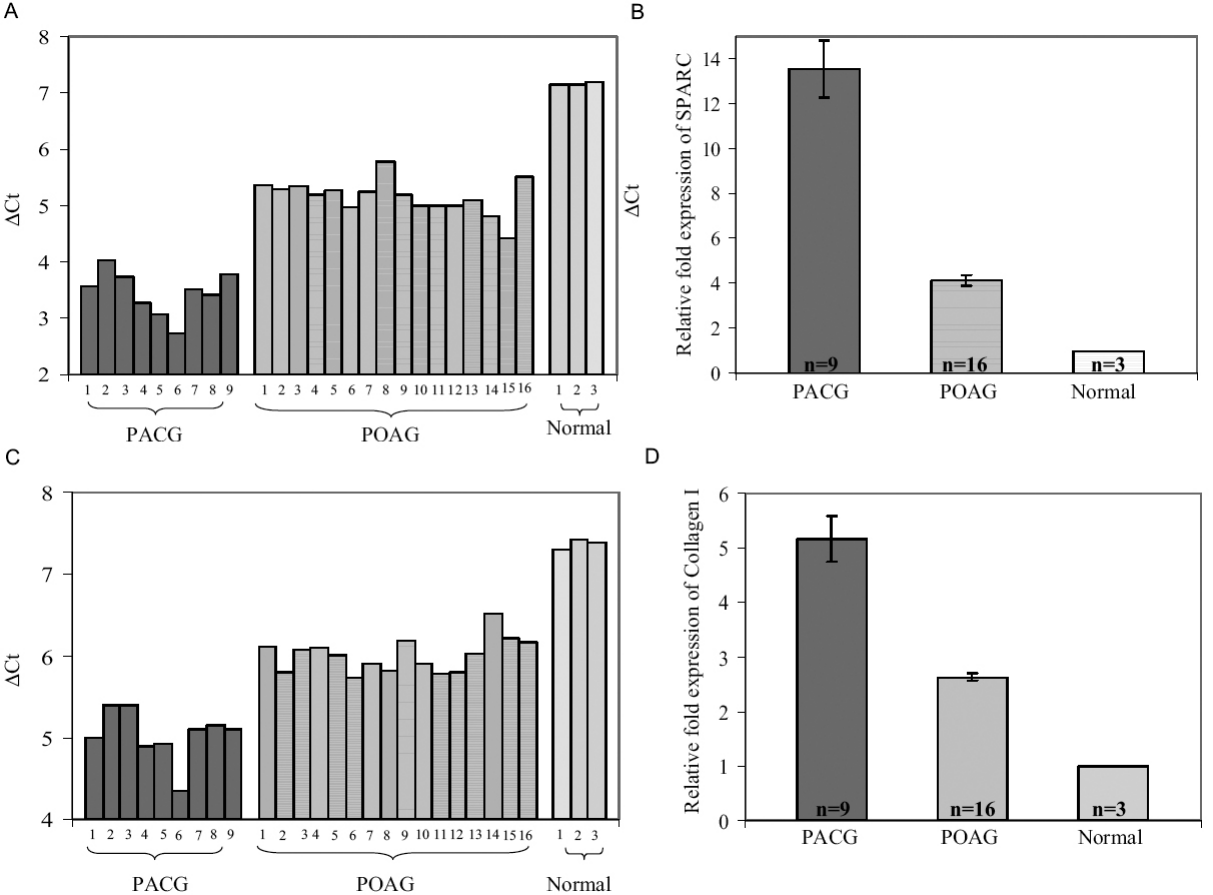
SPARC and collagen I mRNA expression measured by quantitative real-time RT–PCR. Total RNA was purified from the iris stroma of PACG (n=9), POAG (n=16), and normal (n=3) subjects and analyzed by real-time quantitative RT–PCR. **A**: SPARC mRNA expression in PACG, POAG, and non-glaucomatous iris specimens is shown in the chart. The y axis (ΔC_t_) represents SPARC expression normalized against β-actin expression. The data revealed an overexpression of SPARC in the iris tissue from PACG and POAG individuals relative to the iris tissue from non-glaucomatous subjects. **B**: Fold expression of SPARC mRNA in PACG and POAG relative to non-glaucomatous specimens is shown. The data illustrated on the graph represent the mean±SEM of fold expression (2^-∆Ct,PACG/∆Ct,POAG-∆Ct,normal^) of SPARC in PACG and POAG specimens relative to that in non-glaucomatous specimens (fold expression=1). The PACG iris contained a mean 13.6 fold and a mean 3.3 fold more SPARC than non-glaucomatous and POAG iris (p<0.001), respectively. The POAG iris contained a mean 4.1 fold more SPARC than non-glaucomatous iris. **C**: Collagen I mRNA expression in PACG, POAG, and non-glaucomatous iris specimens is shown. The y axis (ΔC_t_) represents collagen I expression normalized against β-actin expression. The data revealed an overexpression of collagen I in the iris from PACG and POAG individuals relative to the iris from normal subjects. **D**: The fold expression of collagen I mRNA in PACG and POAG relative to non-glaucomatous specimens is given in the graph. The data illustrated on the graph represent the mean±SEM of fold expression (2^-∆Ct,PACG/∆Ct,POAG-∆Ct,normal^) of collagen I in PACG and POAG relative to that in non-glaucomatous specimens (fold expression=1). The PACG iris contained a mean 5.2 fold and a mean 2.0 fold more collagen I than non-glaucomatous and POAG iris (p<0.001), respectively, and the POAG iris contained a mean 2.6 fold more collagen I than non-glaucomatous iris.

The expression levels of SPARC and collagen I in PACG, POAG, and non-glaucoma iris specimens were analyzed by real-time RT–PCR. A higher level of SPARC expression in individual iris specimens from PACG eyes compared to both POAG iris specimens and non-glaucoma controls was observed as represented by C_t_ values normalized against the respective β-actin C_t_ values (∆C_t_; Figure 1A). The expression of SPARC was significantly higher in PACG iris specimens with a 3.3 fold and 13.6 fold higher expression than in POAG and non-glaucoma iris specimens, respectively (Figure 1B). A higher level of collagen I expression in individual iris specimens from PACG eyes compared to both POAG iris specimens and non-glaucoma controls was similarly observed (Figure 1C) with PACG iris specimens expressing a mean 2.0 fold and 5.2 fold more collagen I than POAG and non-glaucoma iris specimens, respectively (Figure 1D). These findings provide supporting evidence of a positive relationship between the expression levels of SPARC and collagen I. First, all the PACG iris samples demonstrated a higher expression level of SPARC and collagen I compared to both POAG and non-glaucoma irides. Second, when examined across individual samples, PACG iris specimen number 6 featured an unusually high level of collagen I expression (Figure 1C) and was also associated with a relatively high level of SPARC expression (Figure 1A). Incidentally, specimen number 6 was not from one of the acute angle closure attack eyes.

### Histological analysis of collagen I in iris stroma

**Figure 2:**
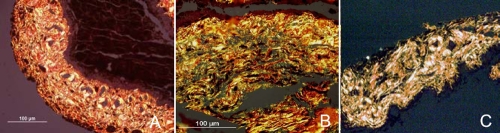
Analysis of iris sections by Sirius red staining. Iris sections from PACG (**A**), POAG (**B**) as well as normal (**C**) patients were stained with Picrosirius red. When viewed with a polarized light, mature type I collagen fibers appear bright yellow or orange. Magnification x20.

Figure 2 shows iris sections stained with picrosirius red stain obtained from PACG, POAG, and non-glaucoma irides. The mean grading for the PACG group was 3.89±0.33 while the mean grading for the POAG group was 2.70±0.52. All three non-glaucoma DSAEK iris specimens demonstrated a grading of 1, which corresponded to the iris grading from a normal fixed eye specimen (data not shown). The difference among the three groups was significant (p<0.001; Table 2) The Bland Altman test showed an excellent agreement of 96% between the two masked independent observers (95% CI 95.9-96.3).

**Table 2:** Histological grading of Picrosirius red staining in iris samples.

**Group**	**n**	**Median grading**	**Mean grading**	**Standard deviation**	**Mann–Whitney U 2-sided**
PACG	9	4	3.89	0.33	<0.001
POAG	16	2	2.7	0.52	<0.001
DSAEK	3	1	1	0	<0.001

The differences in the mean grade between PACG and POAG, PACG and Control, and POAG and Control were significant (p<0.001). n denotes the number of patients in each group.

### Iris stromal blood vessel density

The mean iris stromal blood vessel density per square micron cross-sectional area in the respective groups was as follows: 2.62×0^−5^±1.14 (PACG), 2.97×10^−5^±1.18 (POAG), and 1.99×10^−5^±0.39 (DSAEK, non-glaucomatous iris). There was no significant difference in the density of stromal blood vessels between PACG and POAG, PACG and Control, and POAG and Control groups (Table 3).

**Table 3:** Comparison of mean stromal blood vessel density between PACG and POAG, PACG and non-glaucoma, and POAG and non-glaucoma groups.

	**Groups**	**n**	**mean (SD)**	**Mann–Whitney U p value**
Mean density	PACG	9	2.62 (1.14)	0.50
	POAG	16	2.97 (1.18)	0.50
Mean density	PACG	9	2.62 (1.14)	0.41
	Control	3	1.99 (0.39)	0.41
Mean density	POAG	16	2.97 (1.18)	0.15
	Control	3	1.99 (0.39)	0.15

There was no significant difference in the density of stromal blood vessels between any of the groups compared.

## Discussion

SPARC is a matricellular protein that is secreted by several cell types such as fibroblasts, endothelial cells, and epithelial cells during an inflammatory response, tissue reparative process, and matrix remodeling [25]. One of the principal functions of SPARC is the regulation of collagen I incorporation into tissues. It does so by binding to ECM proteins including collagens I, III, and IV [8], stimulating collagen I production, and cross-linking collagen fibrils to increase tensile strength [19]. This has been clearly demonstrated in the skin of SPARC null-mice, which has been shown to be considerably more fragile than in their wild-type counterparts. Ultrastructural analysis has also revealed that the collagen I fibrils are smaller in diameter in the null species than in wild-type animals [16].

In this study, we found a significant increase in the expression of SPARC in the iris tissue of PACG eyes compared to POAG and non-glaucoma eyes. We also observed a corresponding increase in collagen I deposition in PACG iris. This histological finding of increased deposition of mature collagen I not only concurred with a previous study [7] but also further reinforced the established role of SPARC as a modulator of collagen I production. The marked increase in SPARC, which was detected histologically together with the mRNA data in PACG iris, indicated the presence of a possible ongoing reparative process and/or stress environment. Furthermore, the effect of laser peripheral iridotomy on PACG eyes would exert a local inflammatory effect and account for the observed differences in SPARC and collagen expression between PACG and POAG eyes.

Acute angle closure attacks have been shown to result in significant inflammatory changes to the iris tissue [8], the stress of which could lead to a corresponding increase in SPARC expression and subsequent increase in collagen I cross-linking and reorganization. Interestingly, the majority of PACG eyes in our study did not have any prior history of acute angle closure attacks, particularly PACG patient number 6, whose iris tissue demonstrated the greatest expression of SPARC and collagen I. One possibility for this is that chronic angle closure with PAS or sub-acute angle closure episodes over a period of time may account for the high SPARC levels found in patient number 6. Furthermore, this also suggests that the increased collagen deposition in PACG eyes cannot be entirely accounted for by an acute inflammatory process alone.

Collagens I and III form the majority of the ECM component in the iris stroma [5] with collagen I providing tissue structure and strength. An increase in the content and structural organization of collagen I leads to changes in tissue rigidity. A change in iris rigidity has important influence on its biomechanics, and therefore, iris ECM composition would play an important role in the development of angle closure mechanisms [7]. Furthermore, the development of PAS seen in PACG eyes may be associated in part to the change in the composition of collagen components in the iris ECM, which has been previously reported by He et al. [7]. The authors suggested that the development of PACG might have arisen from a difference in the ratio of collagen I and III composition, which ultimately led to a change in the mechanical response of the tissue and the dynamic relationship between the iris and the trabecular meshwork. In the presence of a narrow or occludable angle, it is reasonable to suggest that changes in the framework of the iris ECM may have a role in the development of PAS and therefore PACG.

An unexpected finding in this study was the notable increase of SPARC in POAG iris (but to a lesser degree than that seen in PACG iris). Chronic use of topical medications, which is often the case with glaucoma patients, potentially leads to a subclinical inflammatory state [26] as a result of the presence of the preservative, benzalkonium chloride, which is known to generate a pro-inflammatory cellular profile [27-29]. Topical prostaglandin analogs have also been reported to disrupt the blood-aqueous barrier [30], hence giving rise to a persistent mild anterior uveitis [31,32]. In both POAG and PACG groups, a comparable mean number of topical glaucoma medications were used, and a similar proportion of patients were on prostaglandin analogs. This pro-inflammatory state induced by prolonged use of topical medications is likely to account for an elevation of SPARC expression observed in both POAG and PACG iris. The significantly greater expression of SPARC in the PACG iris compared to the POAG iris is likely secondary to other factors, which are yet to be determined. Further studies would be necessary to validate our findings.

To determine whether a difference in iris stroma vascularity could be an additional contributory factor for the ultrastructural differences and therefore alteration in the biomechanical properties of the PACG, POAG, and non-glaucoma iris, we devised a simple method to calculate the number of blood vessels and the total surface area of the iris cross-section. Our results showed that there was no difference in the mean density of iris stromal vascularity between PACG, POAG, and non-glaucoma eyes. However, our method of evaluation was subject to several limitations. First, the sampled cross-sectional area of the iris varied with each specimen. The area of each cross-section is dependent upon the size of the tissue obtained during each peripheral iridectomy as well as on the tissue orientation during embedding within paraffin blocks. This issue is exacerbated by the fact that the iridectomy was only a small sampling of the entire iris and that the blood vessels were not likely to be evenly distributed throughout the stroma, hence the larger the area of iris section, the greater probability of blood vessels seen. Second, the apparent number of blood vessel lumen counted in each iris section might have been different cross-sections of the same vessel. Again, this is dependent on both the orientation of the iris tissue during sectioning as well as the inherent orientation of the blood vessels within the stroma, both of which would be difficult to control across all specimens.

A limitation to the study was the control iris used for comparison. It is unproven whether iris from Fuchs eyes would be entirely free from iris damage. However, careful patient selection was conducted to only retrieve iris from patients undergoing DSAEK with a diagnosis of Fuchs without any previous history of intraocular surgery or a history of glaucoma. The use of iris taken from peripheral iridectomies from patients without any other ocular pathology who undergo routine cataract surgery with anterior chamber lens implantation would have been the preferable control, but the number of these patients in the Department is extremely low.

In conclusion, we are the first to demonstrate an increase in SPARC expression in the iris of PACG eyes. POAG eyes also showed elevated SPARC levels albeit to a lesser degree than that observed in PACG eyes. The significance of increased SPARC expression in POAG eyes is yet to be determined. However, in PACG, we propose that SPARC through its effect on iris ECM composition is likely to contribute to changes in iris biomechanics that define angle closure glaucoma. Further work is now necessary to test this hypothesis.
